# Anaerobic Oxidation of Ethane, Propane, and Butane by Marine Microbes: A Mini Review

**DOI:** 10.3389/fmicb.2017.02056

**Published:** 2017-10-23

**Authors:** Rajesh Singh, Michael S. Guzman, Arpita Bose

**Affiliations:** Department of Biology, Washington University in St. Louis, St. Louis, MO, United States

**Keywords:** Gulf of Mexico, short-chain alkanes, sulfate reduction, anaerobic oxidation, *Desulfosarcina/Desulfococcus*

## Abstract

The deep ocean and its sediments are a continuous source of non-methane short-chain alkanes (SCAs) including ethane, propane, and butane. Their high global warming potential, and contribution to local carbon and sulfur budgets has drawn significant scientific attention. Importantly, microbes can use gaseous alkanes and oxidize them to CO_2_, thus acting as effective biofilters. A relative decrease of these gases with a concomitant ^13^C enrichment of propane and *n*-butane in interstitial waters vs. the source suggests microbial anaerobic oxidation. The reported uncoupling of sulfate-reduction (SR) from anaerobic methane oxidation supports their microbial consumption. To date, strain BuS5 isolated from the sediments of Guaymas Basin, Gulf of California, is the only pure culture that can anaerobically degrade propane and *n*-butane. This organism belongs to a metabolically diverse cluster within the *Deltaproteobacteria* called *Desulfosarcina/Desulfococcus*. Other phylotypes involved in gaseous alkane degradation were identified based on stable-isotope labeling and fluorescence *in-situ* hybridization. A novel syntrophic association of the archaeal genus, *Candidatus Syntrophoarchaeum*, and a thermophilic SR bacterium, HotSeep-1 was recently discovered from the Guaymas basin, Gulf of California that can anaerobically oxidize *n*-butane. Strikingly, metagenomic data and the draft genomes of *ca. Syntrophoarchaeum* suggest that this organism uses a novel mechanism for *n*-butane oxidation, distinct from the well-established fumarate addition mechanism. These recent findings indicate that a lot remains to be understood about our understanding of anaerobic SCA degradation. This mini-review summarizes our current understanding of microbial anaerobic SCA degradation, and provides an outlook for future research.

## Introduction

Microbes drive fundamental processes in marine sediments, including the oxidation of organic matter, production of methane and other hydrocarbons, and the removal of sulfate from oceans (Jørgensen, 1982; D'Hondt et al., 2004; Hinrichs et al., 2006; Kallmeyer et al., 2012). The marine biosphere represents a major reservoir for microbial life on Earth. Kallmeyer et al. (2012) estimated the global subseafloor sedimentary microbial abundance to be 2.9 × 10^29^ cells, corresponding to ~0.6% of Earth's total living biomass. Unlike photosynthetic processes, metabolic strategies in dark oceans are based on chemotrophy, where reduced organic and inorganic compounds including methane are the dominant electron donors (Orcutt et al., 2011; Colwell and D'Hondt, 2013). Non-methane short-chain alkanes (SCAs), [ethane (C_2_), propane (C_3_), butane (C_4_; only microbial *n*-butane consumption is noted thus far)] represent additional substrates for primary productivity and play an important role in marine ecosystems. Considerable amounts of SCAs are produced continuously from the ocean via biotic and abiotic processes (0.54 Tg year^−1^ ethane, 0.35 Tg year^−1^ propane, and 0.11 Tg year^−1^ butane) (Plass-Dülmer et al., 1995). Their subsequent escape into the atmosphere significantly contributes to the formation of ozone and organic aerosols (Etiope and Ciccioli, 2009; Pozzer et al., 2010). However, aerobic and anaerobic hydrocarbon-degrading microorganisms can dramatically lower the amount of SCAs reaching the atmosphere (Head et al., 2006; Atlas and Hazen, 2011; Callaghan, 2013). Aerobic microorganisms that can oxidize SCAs are well-characterized (Dworkin and Foster, 1958; Kinnaman et al., 2007; Yakimov et al., 2007; Redmond et al., 2010). Recent studies in cold marine seeps and marine hydrothermal vents have shed light on the microbial anaerobic oxidation of SCAs (Kniemeyer et al., 2007; Adams et al., 2013; Bose et al., 2013; Kleindienst et al., 2014; Dowell et al., 2016; Laso-Pérez et al., 2016). Nevertheless, research on the anaerobic oxidation of ethane is still in its infancy. Microbially mediated anaerobic ethane oxidation linked to SR has been reported for the Gulf of Mexico (GoM) and Middle Valley (MV) sediments (Adams et al., 2013; Bose et al., 2013). However, identification of individual isolates/consortia and the mechanisms involved are still unknown and await discovery. This mini-review summarizes our current understanding of anaerobic microbial SCA degradation, and provides an outlook for future research.

## Short-chain alkanes as carbon and energy source in the marine ecosystems

SCAs are chemically the least reactive compounds due to their non-polar C-H σ-bonds (Carey, 2007). Despite this, microbes can oxidize them aerobically or anaerobically (Leahy and Colwell, 1990; Maeng et al., 1996; Heider et al., 1999; Callaghan et al., 2006; Rojo, 2009; Callaghan, 2013). Aerobes activate alkanes by cleaving C-H bonds via monooxygenase or dioxygenase enzymes (Callaghan et al., 2006). Their terminal oxidation results in an alkanol that is oxidized by dehydrogenases to aldehydes, then to fatty acids followed by β-oxidation (Rabus et al., 2001; Callaghan et al., 2006). The key role that oxygen plays in aerobic alkane transformations led to the belief that alkanes are biologically inert under anoxic conditions. However, research conducted over the years has shown that activation of hydrocarbons can also occur under such conditions. In marine ecosystems, anaerobic SCA degradation is linked to only SR unlike terrestrial ecosystems where nitrate and chlorate act as electron acceptors for hydrocarbon (>C_6_) degradation (Wilkes et al., 2003; Mehboob et al., 2009; Zedelius et al., 2011; Adams et al., 2013; Bose et al., 2013; Kimes et al., 2013; Chanton et al., 2015).

Emission of oil and gas from hydrocarbon seeps are widespread along continental margins. This gas is primarily composed of methane, a potent greenhouse gas; and marine hydrocarbon seeps are estimated to contribute 20 Tg year^−1^ methane to the atmosphere, representing about 5% of the total atmospheric flux (Fung et al., 1991; Judd, 2004). Due to the high concentration of methane in the atmosphere, bacterial oxidation of methane under aerobic and anaerobic conditions has received considerable attention. Over the past four decades, studies focusing on anaerobic oxidation of methane (AOM) have revealed the diversity and distribution of methane oxidizers, and the underlying biochemical processes (Reeburgh, 2007; Knittel and Boetius, 2009; Callaghan, 2013; Haroon et al., 2013). In addition to methane, these hydrocarbon seeps also release an estimated 0.45 Tg year^−1^ ethane and 0.09 Tg year^−1^ propane into the atmosphere (Etiope and Ciccioli, 2009). The amount reaching the atmosphere would be substantially larger if not for microbial oxidation in the sediments and water column (Reeburgh, 2007). Although, microbial aerobic and anaerobic consumption of SCAs from marine and terrestrial environments is widely known (Redmond et al., 2010; Mbadinga et al., 2011; Callaghan, 2013; Musat, 2015), to the best of our knowledge, no quantitative approaches have been used to clearly define the partitioning of SCAs between their atmospheric emission and oxidation. The lack of such data makes it difficult to estimate the influence of SCAs to global carbon budgets and their potential effect on climate. Future research on SCA degradation would help fill this large knowledge gap.

In marine ecosystems, anaerobic oxidation of C_2_-C_4_ alkanes can significantly contribute to community bioenergetics (Lorenson et al., 2002; Formolo et al., 2004; Sassen et al., 2004; Alain et al., 2006; Bose et al., 2013), while competing with AOM for sulfate, an electron acceptor shared by these processes (Joye et al., 2004; Orcutt et al., 2010; Bowles et al., 2011; Adams et al., 2013; Bose et al., 2013). Indeed, at the GoM cold seeps, SR rates are higher than can be accounted for by AOM alone, indicating that SR is potentially linked to the oxidation of non-methane SCAs or higher petroleum hydrocarbons (Joye et al., 2004; Musat et al., 2009; Orcutt et al., 2010; Bowles et al., 2011). The microbial oxidation of SCAs is confirmed by ^13^C-enriched propane and *n*-butane in the sediment interstitial water relative to gas in the hydrates, and the carbonate alkalinity around them (Sassen et al., 2004). Overall, studies on the microbial degradation of SCAs in marine settings are motivated by questions of how such processes affect global carbon and sulfur cycling, and who the participating organisms are.

Anaerobic oxidation of C_2_-C_4_ alkanes with SR has been demonstrated in anoxic, marine settings (Kniemeyer et al., 2007; Savage et al., 2010; Adams et al., 2013; Bose et al., 2013; Kleindienst et al., 2014; Laso-Pérez et al., 2016). These studies have identified novel and metabolically diverse microbes thriving on C_2_-C_4_ alkanes. Sulfate-reducing bacteria (SRB) from the GoM and Guaymas Basin (GB), Gulf of California sediments oxidized C_3_-C_4_ alkanes to CO_2_ (Kniemeyer et al., 2007). These authors tested different temperature regimes (12, 28, and 60°C) using various substrates (methane, ethane, propane, *n*-butane, *iso*-butane, alcohols, or carboxylic acids). A pure culture (BuS5) isolated from 28°C enrichments, anaerobically oxidized C_3_-C_4_ alkanes (Kniemeyer et al., 2007). The strain was determined to be a *Deltaproteobacterium* within the *Desulfosarcina/Desulfococcus* (DSS) cluster. Further, Kleindienst et al. (2014) showed the presence of distinct DSS clades in two seep sediments from the Mediterranean Amon mud volcano and Guaymas Basin degrading *n*-butane and dodecane. Jaekel et al. (2013) enriched microbial populations from the GoM and Hydrate Ridge marine cold seeps that degraded C_3_-C_4_ alkanes. Similar to the previous observation by Kniemeyer et al. (2007), the enrichment cultures degraded propane and *n*-butane simultaneously, but not methane, ethane, *iso*-butane, or pentane. They also identified DSS cluster members as the responsible phylotypes. Using *ex-situ* sediment slurries, Bose et al. (2013) demonstrated the anaerobic oxidation of C_1_-C_4_ alkanes coupled with SR. Interestingly, these authors observed ethane consumption comparable to methane, propane, and *n*-butane. This is in contrast to the study by Kniemeyer et al. (2007), who reported extremely slow rates of ethane-driven SR. A notable difference between these studies is the use of sediment slurries by Bose et al. (2013) compared to enrichment techniques used by Kniemeyer et al. (2007). Community analyses suggested the enhancement of *Deltaproteobacteria* in SCA amended reactors. Deltaproteobacterial sequences from ethane incubations were closely related to the isolate BuS5, and the enrichment culture Butane 12-GMe (both isolated from marine sediments by Kniemeyer et al., 2007 and Bose et al., 2013).

Anaerobic SCA degradation was also demonstrated in metalliferous, organic-poor Middle Valley hydrothermal vent sediments at 25, 55, and 75°C. Sediment slurries showed degradation of C_1_-C_4_ alkanes under SR conditions (Adams et al., 2013). Comparison of bacterial communities, suggested the presence of *Deltaproteobacteria* mediating the anaerobic oxidation of C_1_-C_4_ alkanes. This implied that, anaerobic alkane degraders exist in both cold marine seeps and high temperature hydrothermal vent systems. Importantly, anaerobic oxidation of SCAs is not restricted to only the *Deltaproteobacteria*. For example, an enrichment from GB sediments with propane at 60°C was dominated by Gram positive, SRB closely related to *Desulfotomaculum*, a commonly found cluster of bacteria in the subsurface biosphere within the *Peptococcaceae* (Ollivier et al., 2007; Wang et al., 2008; Aüllo et al., 2013).

The diversity of SCA degraders in marine environments was recently demonstrated by the discovery of syntrophic *n*-butane degraders from a thermophilic enrichment culture from the GB vent area (Laso-Pérez et al., 2016). Syntrophic association of the archaeal genus, *Candidatus Syntrophoarchaeum* and a thermophilic SRB, HotSeep-1 completely oxidized *n*-butane to CO_2_. A subsequent study on GB hydrothermal mound sediments used bacterial and archaeal 16S rRNA gene clone libraries and V6 tag pyrosequencing to show the co-occurrence of archaeal groups (such as, anaerobic methane-oxidizing archaea ANME-1, ANME-1Guaymas, and ANME-2) with bacterial groups (such as, SEEP-SRB2 and HotSeep-1) (Dowell et al., 2016). This corroborates that an archaeal-bacterial syntrophic community mediates alkane degradation in a GB hydrothermal mound.

The HotSeep-1 group was also detected in thermophilic SR enrichments with *n*-butane from GB at 60°C (Kniemeyer et al., 2007), and in SR enrichments inoculated with MV hydrothermal sediments amended with C_2_-C_4_ alkanes at 55°C (Adams et al., 2013). It is likely that the organisms in the HotSeep-1 group do not oxidize these alkanes directly but function as versatile syntrophs that serve as electron/hydrogen sinks within different consortia (Zengler et al., 1999; Dowell et al., 2016). Together, these findings reflect the diversity of anaerobic microorganisms thriving on non-methane SCAs in marine environments. These studies also suggest that the processes mediated by these organisms contribute to ocean chemistry and community bioenergetics via both sulfate and SCA removal.

Despite informative studies over the past decade on anaerobic SCA degradation, very little is known about ethane-oxidizing phylotypes, and how they interact with other organisms in deep-sea ecosystems. Kniemeyer et al. (2007) reported ethane dependent SR in an enrichment from the GoM at 12°C. However, the reported rate was orders of magnitude slower than the oxidation rates of C_3_-C_4_ alkanes. Notably, Bose et al. (2013) observed ethane consumption approximately two orders of magnitude higher than those reported by Kniemeyer et al. (2007) in *ex-situ* slurry incubations of GoM sediments incubated at 7°C. It is possible that SRB closely related to the C_3_-C_4_ degrading DSS cluster might be associated with ethane degradation in these incubations, though this remains to be investigated. These authors also demonstrated carbon flux dynamics of ethane oxidation using δ^13^C of DIC and alkanes from their enrichment experiments.

Batch incubations with sediments from MV hydrothermal vent systems showed modest rates of ethane dependent SR at 25, 55, and 75°C (Adams et al., 2013). In accordance with the observed stoichiometries, SR coupled to the anaerobic oxidation of C_2_-C_4_ proceeded at a faster rate than AOM at mesophilic and thermophilic temperatures (25 and 55°C, respectively). These faster rates might indirectly limit AOM. These findings suggest that microbial anaerobic SCA degradation affects local carbon and sulfur cycles. In contrast to C_3_-C_4_ alkane degradation, anaerobic ethane oxidation has not yet been reported by sediment-free cultures or isolates/consortia. This makes ethane oxidation the least understood among the SCA degradative processes. This is in part due to the slow rate of microbial anaerobic ethane oxidation observed in enrichment studies (Kniemeyer et al., 2007). Anaerobic ethane oxidation needs to be investigated in further detail due to its abundance in marine seeps (Plass-Dülmer et al., 1995; Sassen et al., 1998; Etiope and Ciccioli, 2009), its contribution to tropospheric chemistry (Singh et al., 1994; Katzenstein et al., 2003), and the demonstrated ability of microbial populations to degrade ethane linked to SR (Adams et al., 2013; Bose et al., 2013). Studies should focus on the precise nature and extent of this process; the responsible microbes; and the associated biochemistry.

## Biochemistry of anaerobic short-chain alkane oxidation

**Figure 1:**
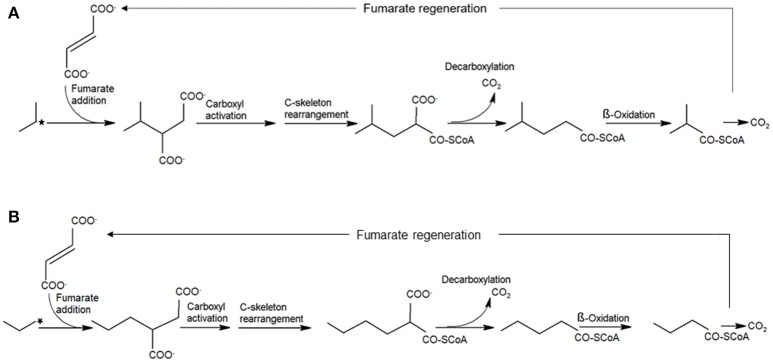
Anaerobic activation of propane at the sub-terminal **(A)** and terminal **(B)** carbon atom (marked with stars) via fumarate addition yielding isopropylsuccinate and *n*-propylsuccinate, respectively. A similar activation mechanism exclusively at the sub-terminal carbon atom is proposed for the anaerobic oxidation of *n*-butane.

Fumarate addition is noted as the biochemical mechanism for aromatic hydrocarbon and *n*-alkane activation by anaerobes (Biegert et al., 1996; Beller and Spormann, 1997; Kropp et al., 2000; Rabus et al., 2001; Wilkes et al., 2002; Callaghan et al., 2006, 2008, 2012; Grundmann et al., 2008). In this pathway, *n*-alkanes are activated by fumarate addition to the double bond at the sub-terminal or terminal carbon producing 2-(1-methylalkyl)succinates (or 2-alkylsuccinates). Degradation of 2-(1-methylalkyl)succinates involves carbon skeleton rearrangement, and decarboxylation yielding branched fatty acids followed by β-oxidation (Widdel and Grundmann, 2010; Agrawal and Gieg, 2013; Callaghan, 2013; Musat, 2015) (Figure 1). C_3_-C_4_ alkane activation by strain BuS5 and, in the marine enrichment Propane60-GuB is suggested to occur via the same pathway (Kniemeyer et al., 2007). Based on the metabolites detected in both cultures, activation of *n*-butane presumably occurs at the secondary carbon yielding (1-methylpropyl) succinate. Interestingly, it was suggested that propane activation occurs at both secondary and primary carbon atoms producing isopropyl- and *n*-propylsuccinate respectively (Kniemeyer et al., 2007) (Figure 1). Although initially considered a side reaction, the second pathway was substantiated by incubations of strain BuS5 with position-specific deuterium-labeled propane (Jaekel et al., 2014). Results showed that the activation of propane at the secondary carbon is more significant, accounting for an estimated 70% of the activation events, with 30% of activation occurring at the primary carbon. Based on these findings, activation of ethane would likely yield ethylsuccinate. Although, metabolites analyses in ethane-degrading laboratory batch reactors are lacking, ethylsuccinate is reported from hydrocarbon-rich settings such as, in crude oil processing facilities and production wells (Duncan et al., 2009), oilfields (Gieg et al., 2010), and coal beds (Wawrick et al., 2012).

At the biochemical level, this process involves the abstraction of an H atom from the alkane substrate by the glycyl radical enzyme (GRE), 1-methylalkyl succinate synthase (MAS) (Grundmann et al., 2008) also known as alkylsuccinate synthase (ASS) (Callaghan et al., 2008). These enzymes utilize free radicals to catalyze fumarate addition to form a succinate radical intermediate (Callaghan et al., 2008, 2010; Bharadwaj et al., 2015; Musat, 2015). Several PCR-based detection assays target the genes that encode the α-subunit of the MAS/ASS enzyme (*masD/assA*) as the most relevant genetic markers for anaerobic alkane degradation by fumarate addition (Callaghan et al., 2010; Aitken et al., 2013; Von Netzer et al., 2013; Gittel et al., 2015).

**Figure 2:**
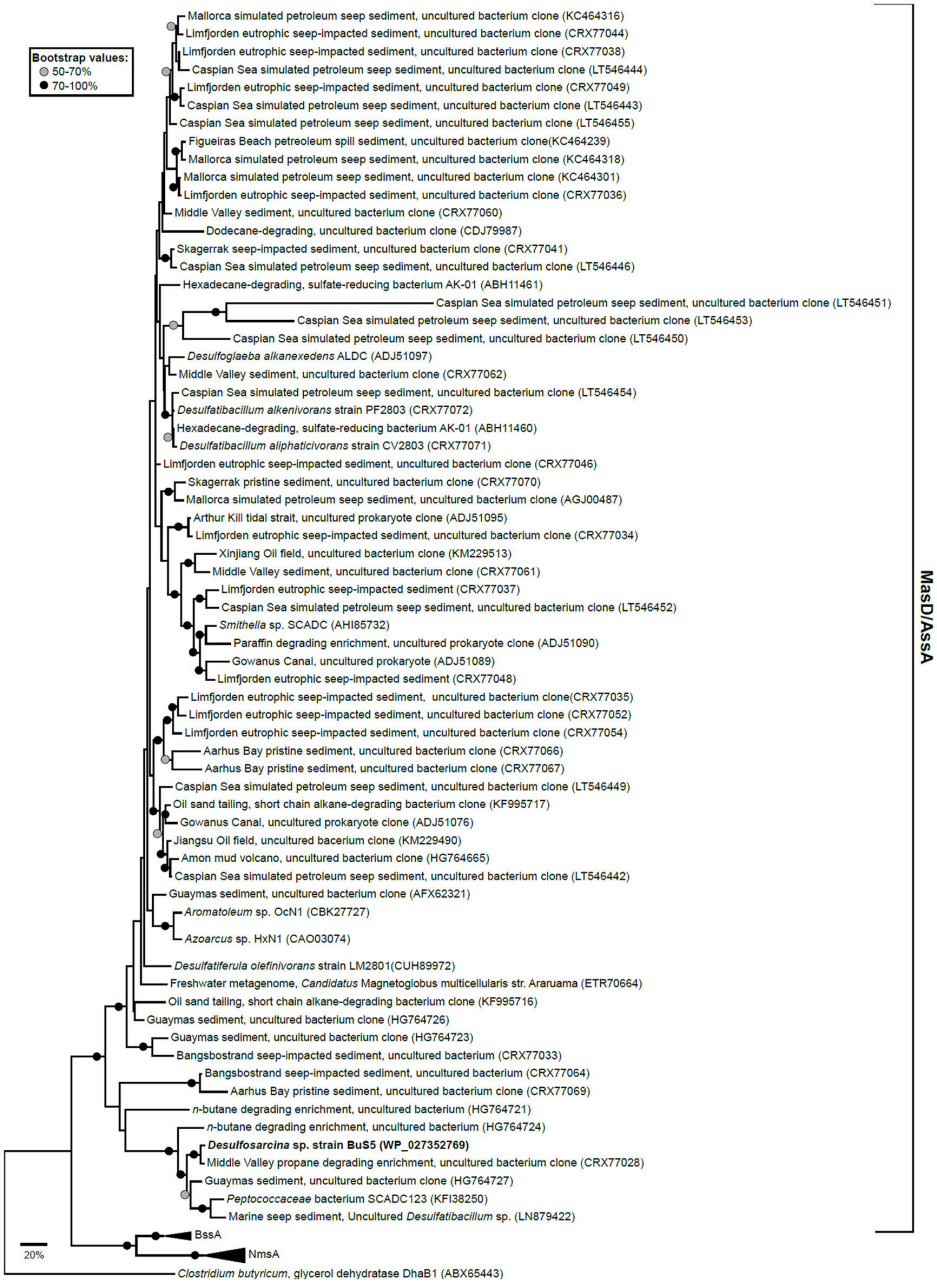
Maximum likelihood tree of translated full-length and partial *masD/assA/bssA/nmsA* homologs from selected isolates as well as pristine and seepage-impacted metagenomes obtained from GenBank (accession numbers are shown in parentheses). Tree was inferred using the Le_Gascuel_2008 model (Le and Gascuel, 2008) and involved 85 amino acid sequences and a total of 210 positions. All positions with less than 95% site coverage were eliminated. Full-length glycerol dehydratase (*dhaB1*) from *Clostridium butyricum* was used as an outgroup. Node circles denote bootstrap value percentages from 100 replicate trees. Scale bar represents 20% estimated sequence divergence. Evolutionary tree was constructed in MEGA7 (Kumar et al., 2016).

The genes encoding an alkane activating GRE have been identified in the SRB *Desulfatibacillum alkenivorans* AK-01 (Callaghan et al., 2008) and *Desulfoglaeba alkanexedens* ALDCT (Callaghan et al., 2010) and in nitrate reducing strains HxN1 (Grundmann et al., 2008) and OcN1 (Zedelius et al., 2011), all affiliated to the *Deltaproteobacteria*. Recent genome analysis of strain BuS5 identified a single putative *masD* gene, suggesting that one MasD is involved in the activation of both propane and *n*-butane (Musat, 2015). Phylogenetic reconstruction of translated full-length and partial *masD/assA/bssA/nmsA* homologs from selected isolates, as well as pristine and seepage-impacted metagenomes showed that there is a wide diversity of organisms that can degrade short-, long-chain hydrocarbons, and cyclic aromatic hydrocarbons (Figure 2). Gittel et al. (2015) designed novel PCR primers to the *masD/assA* gene to determine the diversity and distribution of anaerobic alkane degraders in pristine and seepage-impacted Danish coastal sediments. Seepage-impacted sediments were dominated by a single *masD/assA* gene cluster, which indicates an occurrence of a substrate-adapted community. In contrast, pristine sediments harbored a diverse range of *masD/assA* phylotypes including those present in seepage-impacted sediments. This comprehensive cultivation-independent survey of the diversity and distribution of anaerobic alkane degraders highlighted the relevance of *masD/assA* genes as diagnostic genetic markers to identify seepage/microseepage, e.g., during oil and gas prospecting, and may act as an indicator of anthropogenic oil spills in marine sediments. Stagars et al. (2016) used *masD* to study the diversity of alkane degraders from seven globally distributed marine seeps. They identified three distinct *masD* clades, indicating a high number of anaerobic alkane degraders thriving in such environments. Recently, a novel syntrophic association for *n*-butane oxidation by the archaeal genus, *Candidatus Syntrophoarchaeum* and a thermophilic SRB, HotSeep-1 was proposed in an anaerobic thermophilic enrichment from the GB vent area (Laso-Pérez et al., 2016). Neither the metagenomics assembly nor the draft genomes of *ca*. *Syntrophoarchaeum* contained any GRE indicating a different mechanism for *n*-butane activation. Butane activation is proposed to occur via a methyl-coenzyme M reductase (MCR) enzyme analogous to that of the MCR in AOM. This is supported by the detection of butyl-coenzyme M as a reaction intermediate in cell extracts. The reducing equivalents are then presumably transferred to strain HotSeep-1.

## Summary and future research directions in microbial short-chain alkane degradation

Microbial anaerobic oxidation of C_2_-C_4_ alkanes has received considerable scientific attention in recent years. Research efforts have helped shed light on key questions such as the degradative microbes, the pathways underlying the degradation, the enzymes involved, and the potential influence of anaerobic SCA degradation on local carbon and sulfur cycling. Based on geochemical and microbiological studies from cold hydrocarbon seeps to hydrothermal vents, it appears that microbes that degrade propane and *n*-butane are more easily enriched than ethane despite its high concentrations in marine ecosystems. Most of the SCA degraders discovered *in-situ* or *ex-situ* batch experiments are within the *Desulfosarcina-Desulfococcus* cluster of the *Deltaproteobacteria*, although a syntrophic community has been discovered recently. Although scientists have made significant progress in this field by isolating propane and *n*-butane degrading strains, our understanding of microbial anaerobic oxidation of ethane is still in its early stages. Thus, an obvious need to understand this process in marine ecosystems exists. Further, investigations into anaerobic SCA degradation could possibly point to novel and unknown degradative processes that can potentially strongly influence the carbon and sulfur biogeochemical cycles.

## Author contributions

RS performed all the necessary literature searches and data compilation. MG performed the phylogenetic analysis. RS and AB wrote the manuscript.

### Conflict of interest statement

The authors declare that the research was conducted in the absence of any commercial or financial relationships that could be construed as a potential conflict of interest.

## References

[B1] Adams M. M. Hoarfrost A. L. Bose A. Joye S. B. Girguis P. R. (2013). Anaerobic oxidation of short-chain alkanes in hydrothermal sediments: potential influences on sulfur cycling and microbial diversity. Front. Microbiol. 4:110. 10.3389/fmicb.2013.00110 23717305 PMC3653109

[B2] Agrawal A. Gieg L. M. (2013). *In situ* detection of anaerobic alkane metabolites in subsurface environments. Front. Microbiol. 4:140. 10.3389/fmicb.2013.00140 23761789 PMC3671572

[B3] Aitken C. M. Jones D. M. Maguire M. J. Gray N. D. Sherry A. Bowler B. F. J. (2013). Evidence that crude oil alkane activation proceeds by different mechanisms under sulfate-reducing and methanogenic conditions. Geochim. Cosmochim. Acta 109, 162–174. 10.1016/j.gca.2013.01.031

[B4] Alain K. Holler T. Musat F. Elvert M. Treude T. Krüger M. (2006). Microbiological investigation of methane-and hydrocarbon-discharging mud volcanoes in the Carpathian Mountains, Romania. Environ. Microbiol. 8, 574–590. 10.1111/j.1462-2920.2005.00922.x 16584470

[B5] Atlas R. M. Hazen T. C. (2011). Oil biodegradation and bioremediation: a tale of the two worst spills in U.S. history. Environ. Sci. Technol. 45, 6709–6715. 10.1021/es2013227 21699212 PMC3155281

[B6] Aüllo T. Ranchou-Peyruse A. Ollivier O. Magot M. (2013). *Desulfotomaculum* spp. and related gram-positive sulfate-reducing bacteria in deep subsurface environments. Front. Microbiol. 4:632. 10.3389/fmicb.2013.00362 24348471 PMC3844878

[B7] Beller H. R. Spormann A. (1997). Anaerobic activation of toluene and o-xylene by addition to fumarate in denitrifying strain T. J. Bacteriol. 179, 670–676. 10.1128/jb.179.3.670-676.1997 9006019 PMC178746

[B8] Bharadwaj V. S. Vyas S. Villano S. M. Maupin C. M. Dean A. M. (2015). Unravelling the impact of hydrocarbon structure on the fumarate addition mechanism-a gas-phase ab initio study. Phys. Chem. Chem. Phys. 17, 4054–4066. 10.1039/C4CP04317K 25566585

[B9] Biegert T. Fuchs G. Heider J. (1996). Evidence that anaerobic oxidation of toluene in the denitrifying bacterium *Thauera aromatica* is initiated by formation of benzylsuccinate from toluene and fumarate. Eur. J. Biochem. 238, 661–668. 10.1111/j.1432-1033.1996.0661w.x 8706665

[B10] Bose A. Rogers D. R. Adams M. M. Joye S. B. Girguis P. R. (2013). Geomicrobiological linkages between short-chain alkane consumption and sulfate reduction rates in seep sediments. Front. Microbiol. 4:386. 10.3389/fmicb.2013.00386 24376442 PMC3860272

[B11] Bowles M. W. Samarkin V. A. Bowles K. M. Joye S. B. (2011). Weak coupling between sulfate reduction and the anaerobic oxidation of methane in methane-rich seafloor sediments during *ex situ* incubation. Geochim. Cosmochim. Acta 75, 500–519. 10.1016/j.gca.2010.09.043

[B12] Callaghan A. V. (2013). Enzymes involved in the anaerobic oxidation of n-alkanes from methane to long-chain paraffins. Front. Microbiol. 4:89. 10.3389/fmicb.2013.00089 23717304 PMC3653055

[B13] Callaghan A. V. Davidova I. A. Savage-Ashlock K. Parisi V. A. Gieg L. M. Suflita J. M.. (2010). Diversity of benzyl- and alkylsuccinate synthase genes in hydrocarbon-impacted environments and enrichment cultures. Environ. Sci. Technol. 44, 7287–7294. 10.1021/es1002023 20504044

[B14] Callaghan A. V. Gieg L. M. Kropp K. G. Suflita J. M. Young L. Y. (2006). Comparison of mechanisms of alkane metabolism under sulfate-reducing conditions among two bacterial isolates and a bacterial consortium. Appl. Environ. Microbiol. 72, 4274–4282. 10.1128/AEM.02896-05 16751542 PMC1489600

[B15] Callaghan A. V. Morris B. E. Pereira I. A. McInerney M. J. Austin R. N. Groves J. T.. (2012). The genome sequence of *Desulfatibacillum alkenivorans* AK-01: a blueprint for anaerobic alkane oxidation. Environ. Microbiol. 14, 101–113. 10.1111/j1462-2920.2011.02516.x 21651686

[B16] Callaghan A. V. Wawrik B. Ní Chadhain S. M. Young L. Y. Zylstra G. J. (2008). Anaerobic alkane degrading strain AK-01 contains two alkylsuccinate synthase genes. Biochem. Biophys. Res. Commun. 366, 142–148. 10.1016/j.bbrc.2007.11.094 18053803

[B17] Carey F. A. (2007). Organic Chemistry, 7th Edn. New York, NY: McGraw-Hill Science, 1312.

[B18] Chanton J. Zhao T. Rosenheim B. E. Joye S. Bosman S. Brunner C.. (2015). Using natural abundance radiocarbon to trace the flux of petrocarbon to the seafloor following the deepwater horizon oil spill. Environ. Sci. Technol. 49, 847–854. 10.1021/es5046524 25494527

[B19] Colwell F. S. D'Hondt S. (2013). Nature and extent of deep biosphere. Rev. Miner. Geochem. 45, 547–574. 10.2138/rmg.2013.75.17

[B20] D'Hondt S. Jørgensen B. B. Miller D. J. Batzke A. Blake R. Cragg B. A.. (2004). Distributions of microbial activities in deep subseafloor sediments. Science 306, 2216–2221. 10.1126/science.1101155 15618510

[B21] Dowell F. Cardman Z. Dasarathy S. Kellermann M. Y. Lipp J. S. Ruff S. E.. (2016). Microbial communities in ethane-and short chain alkane-rich hydrothermal sediments of guaymas basin. Front. Microbiol. 7:17. 10.3389/fmicb.2016.00017 26858698 PMC4731509

[B22] Duncan K. E. Gieg L. M. Parisi V. A. Tanner R. S. Tringe S. G. Bristow J.. (2009). Biocorrosive thermophilic microbial communities in Alaskan North Slope oil facilities. Environ. Sci. Technol. 43, 7977–7984. 10.1021/es9013932 19921923

[B23] Dworkin M. Foster J. W. (1958). Experiments with some microorganisms which utilize ethane and hydrogen. J. Bacteriol. 75, 592–603. 13538930 10.1128/jb.75.5.592-603.1958 PMC290115

[B24] Etiope G. Ciccioli P. (2009). Earth's degassing: a missing ethane and propane source. Science 323:478. 10.1126/science.1165904 19164741

[B25] Formolo M. J. Lyons T. W. Zhang C. Kelley C. Sassen R. Horita J. (2004). Quantifying authigenic carbonates at gas hydrate sites in the Gulf of Mexico. Chem. Geol. 205, 253–264. 10.1016/j.chemgeo.2003.12.021

[B26] Fung I. Prather M. John J. Lerner J. Matthews E. (1991). Three dimensional model synthesis of the global methane cycle. J. Geophys. Res. 96, 13033–13065. 10.1029/91JD01247

[B27] Gieg L. M. Davidova I. A. Duncan K. E. Suflita J. M. (2010). Methanogenesis, sulfate reduction and crude oil biodegradation in hot Alaskan oilfields. Environ. Microbiol. 12, 3074–3086. 10.1111/j.1462-2920.2010.02282.x 20602630

[B28] Gittel A. Donhauser J. Røy H. Girguis P. R. Jørgensen B. B. Kjeldsen K. U. (2015). Ubiquitous presence and novel diversity of anaerobic alkane degraders in cold marine sediments. Front. Microbiol. 6:1414. 10.3389/fmicb.2015.01414 26733961 PMC4681840

[B29] Grundmann O. Behrends A. Rabus R. Amann J. Halder T. Heider J.. (2008). Genes encoding the candidate enzyme encoding for anaerobic activation of n-alkanes in the denitrifying bacterium, strain HxN1. Environ. Microbiol. 10, 376–385. 10.1111/j.1462-2920.2007.01458.x 17961174

[B30] Haroon M. F. Hu S. Shi Y. Imelfort M. Keller J. Hugenholtz P.. (2013). Anaerobic oxidation of methane coupled to nitrate reduction in a novel archaeal lineage. Nature 500, 567–570. 10.1038/nature12375 23892779

[B31] Head I. M. Jones D. M. Röling W. F. (2006). Marine microorganisms make a meal of oil. Nat. Rev. Microbiol. 4, 173–182. 10.1038/nrmicro1348 16489346

[B32] Heider J. Spormann A. M. Beller H. R. Widdel F. (1999). Anaerobic bacterial metabolism of hydrocarbons. FEMS Microbiol. Rev. 22, 459–473.

[B33] Hinrichs K.-U. Hayes J. M. Bach W. Spivack A. J. Hmelo L. R. Holm N. G.. (2006). Biological formation of ethane and propane in the Deep marine subsurface. Proc. Natl. Acad. Sci. U.S.A. 103, 14684–14689. 10.1073/pnas.0606535103 16990430 PMC1595412

[B34] Jaekel U. Musat N. Adam B. Kuypers M. Grundmann O. Musat F. (2013). Anaerobic degradation of propane and butane by sulfate-reducing bacteria enriched from marine hydrocarbon cold seeps. ISME J. 7, 885–895. 10.1038/ismej.2012.159 23254512 PMC3635235

[B35] Jaekel U. Vogt C. Fischer A. Richnow H.-H. Musat F. (2014). Carbon and hydrogen stable isotope fractionation associated with the anaerobic degradation of propane and butane by marine sulfate-reducing bacteria. Environ. Microbiol. 16, 130–140. 10.1111/1462-2920.12251 24028539

[B36] Jørgensen B. B. (1982). Mineralization of organic matter in the sea bed—the role of sulphate reduction. Nature 296, 643–645. 10.1038/296643a0

[B37] Joye S. B. Boetius A. Orcutt B. N. Montoya J. P. Schulz H. N. Erickson M. J. (2004). The anaerobic oxidation of methane and sulfate reduction in sediments from Gulf of Mexico cold seeps. Chem. Geol. 25, 219–238. 10.1016/j.chemgeo.2003.12.019

[B38] Judd A. (2004). Natural seabed gas seeps as sources of atmospheric methane. Environ. Geol. 46, 988–996. 10.1007/s00254-004-1083-3

[B39] Kallmeyer J. Pockalny R. Adhikari R. R. Smith D. C. D'Hondt S. (2012). Global distribution of microbial abundance and biomass in subseafloor sediment. Proc. Natl. Acad. Sci. U.S.A. 109, 16213–16216. 10.1073/pnas.1203849109 22927371 PMC3479597

[B40] Katzenstein A. S. Doezema L. A. Simpson I. J. Blake D. R. Rowland F. S. (2003). Extensive regional atmospheric hydrocarbon pollution in the southwestern United States. Proc. Natl. Acad. Sci. U.S.A. 100, 11975–11979. 10.1073/pnas.1635258100 14530403 PMC218698

[B41] Kimes N. E. Callaghan A. V. Aktas D. F. Smith W. L. Sunner J. Golding B.. (2013). Metagenomic analysis and metabolite profiling of deep-sea sediments from the Gulf of Mexico following the Deepwater Horizon oil spill. Front. Microbiol. 4:50. 10.3389/fmicb.2013.00050 23508965 PMC3598227

[B42] Kinnaman F. S. Valentine D. L. Tyler S. C. (2007). Carbon and hydrogen isotope fractionation associated with the aerobic microbial oxidation of methane, ethane, propane and butane. Geochim. Cosmochim. Acta 71, 271–283. 10.1016/j.gca.2006.09.007.

[B43] Kleindienst S. Herbst F.-A. Stagars M. Netzer F. V. Bergen M. V. Seifert J.. (2014). Diverse sulfate-reducing bacteria of the *Desulfosarcina/Desulfococcus* clade are the key alkane degraders at marine seeps. ISME J. 8, 2029–2044. 10.1038/ismej.2014.51 24722631 PMC4184016

[B44] Kniemeyer O. Musat F. Sievert S. M. Knittel K. Wilkes H. Blumenburg M.. (2007). Anaerobic oxidation of short-chain hydrocarbons by marine sulphate-reducing bacteria. Nature 449, 898–902. 10.1038/nature06200 17882164

[B45] Knittel K. Boetius A. (2009). Anaerobic oxidation of methane: progress with an unknown process. Annu. Rev. Microbiol. 63, 311–334. 10.1146/annurev.micro.61.080706.093130 19575572

[B46] Kropp K. G. Davidova I. A. Suflita J. M. (2000). Anaerobic oxidation of n-dodecane by an addition reaction in a sulfate-reducing bacterial enrichment culture. Appl. Environ. Microbiol. 66, 5393–5398. 10.1128/AEM.66.12.5393-5398.2000 11097919 PMC92473

[B47] Kumar S. Stecher G. Tamura K. (2016). MEGA7: molecular evolutionary genetics analysis version 7.0 for bigger datasets. Mol. Biol. Evol. 33, 1870–1874. 10.1093/molbev/msw054 27004904 PMC8210823

[B48] Laso-Pérez R. Wagener G. Knittel K. Widdel F. Harding K. J. Krukenberg V.. (2016). Thermophilic archaea activate butane via alkyl-coenzyme M formation. Nature 539, 396–401. 10.1038/nature20152 27749816

[B49] Le S. Q. Gascuel O. (2008). An improved general amino acid replacement matrix. Mol. Biol. Evol. 25, 1307–1320. 10.1093/molbev/msn067 18367465

[B50] Leahy J. G. Colwell R. R. (1990). Microbial degradation of hydrocarbons in the environment. Microbiol. Rev. 54, 305–315. 2215423 10.1128/mr.54.3.305-315.1990 PMC372779

[B51] Lorenson T. D. Kvenvolden K. A. Hostettler F. D. Rosenbauer R. J. Orange D. L. Martin J. B. (2002). Hydrocarbon geochemistry of cold seeps in the Monterey Bay National Marine Sanctuary. Mar. Geol. 181, 285–304. 10.1016/S0025-3227(01)00272-9

[B52] Maeng J. H. Sakai Y. Tani Y. Kato N. (1996). Isolation and characterization of a novel oxygenase that catalyzes the first step of n-alkane oxidation in Acinetobacter sp. strain M-1. J. Bacteriol. 178, 3695–3700. 10.1128/jb.178.13.3695-3700.1996 8682768 PMC178149

[B53] Mbadinga S. M. Wang L.-Y. Zhou L. Liu J. E. Gu J.-D. Mu B.-Z. (2011). Microbial communites involved in anaerobic degradation of alkanes. Int. Biodeter. Biodeg. 65, 1–13. 10.1016/j.ibiod.2010.11.009

[B54] Mehboob F. Junca H. Schraa G. Stams A. (2009). Growth of *Pseudomonas chloritidismutans* AW-1T on n-alkanes with chlorate as electron acceptor. Appl. Microbiol. Biotechnol. 83, 739–747. 10.1007/s00253-009-1985-9 19352644 PMC2690828

[B55] Musat F. (2015). The anaerobic degradation of gaseous, nonmethane alkanes-from *in situ* processes to microorganisms. Comput. Struct. Biotechnol. J. 13, 222–228. 10.1016/j.csbj.2015.03.002 25904994 PMC4402382

[B56] Musat F. Galushko A. Jacob J. Widdel F. Kube M. Reinhardt R.. (2009). Anaerobic degradation of naphthalene and 2-methylnaphthalene by strains of marine sulfate-reducing bacteria. Environ. Microbiol. 11, 209–219. 10.1111/j.1462-2920.2008.01756.x 18811643

[B57] Ollivier B. Cayol J.-L. Fauque G. (2007). Sulphate-reducing bacteria from oil fields environments and deep-sea hydrothermal vents, in Sulphate-Reducing Bacteria: Environmental and Engineered Systems, eds Barton L. Hamilton W. (London: Cambridge University Press), 305–328.

[B58] Orcutt B. N. Joye S. B. Kleindienst S. Knittel K. Ramette A. Reitz A. (2010). Impact of natural oil and higher hydrocarbons on microbial diversity, distribution, and activity in Gulf of Mexico cold-seep sediments. Deep Sea Res. II Top. Stud. Oceanogr. 57, 2008–2021. 10.1016/j.dsr2.2010.05.014

[B59] Orcutt B. N. Sylvan J. B. Knab N. J. Edwards K. J. (2011). Microbial Ecology of the Dark Ocean above, at, and below the Seafloor. Microbiol. Mol. Biol. Rev. 75, 361–422. 10.1128/MMBR.00039-10 21646433 PMC3122624

[B60] Plass-Dülmer C. Koppmann R. Ratte M. Rudolf J. (1995). Light non methane hydrocarbon in sea water. Global Geochem. Cycles 9, 79–100. 10.1029/94GB02416

[B61] Pozzer A. Pollmann J. Taraborrelli D. Jöckel P. Helmig D. Tans P. (2010). Observed and simulated global distribution and budget of atmospheric C2-C5 alkanes. Atmos. Chem. Phys. 10, 4403–4422. 10.5194/acp-10-4403-2010

[B62] Rabus R. Wilkes H. Behrends A. Armstroff A. (2001). Anaerobic initial reaction of n-alkanes in a denitrifying bacterium: evidence for (1-methylpentyl)succinate as initial product and for involvement of an organic radical in n-hexane metabolism. J. Bacteriol. 183, 1707–1715. 10.1128/JB.183.5.1707-1715.2001 11160102 PMC95056

[B63] Redmond M. C. Valentine D. L. Sessions A. L. (2010). Identification of novel methane-, ethane-, and propane oxidizing bacteria at marine hydrocarbon seeps by stable isotope probing. Appl. Environ. Microbiol. 76, 6412–6422. 10.1128/AEM.00271-10 20675448 PMC2950463

[B64] Reeburgh W. S. (2007). Oceanic methane biogeochemistry. Chem. Rev. 107, 486–513. 10.1021/cr050362v 17261072

[B65] Rojo F. (2009). Degradation of alkanes by bacteria. Environ. Microbiol. 11, 2477–2490. 10.1111/j.1462-2920.2009.01948.x 19807712

[B66] Sassen R. Macdonald I. R. Guinasso N. L. Joye S. Requejo A. G. Sweet S. T. (1998). Bacterial methane oxidation in sea-floor gas hydrate: significance to life in extreme environments. Geology 26, 851–854. 10.1130/0091-7613(1998)026<0851:BMOISF>2.3.CO;2

[B67] Sassen R. Roberts H. H. Carney R. Milkov A. V. De Freitas D. A. Lanoil B. (2004). Free hydrocarbon gas, gas hydrate, and authigenic Minerals in chemo synthetic communities of the northern Gulf of Mexico Continental slope: relation to microbial processes. Chem. Geol. 205, 195–217. 10.1016/j.chemgeo.2003.12.032

[B68] Savage K. N. Krumholz L. R. Gieg L. M. Parisi V. A. Suflita J. M. Allen J.. (2010). Biodegradation of low-molecular-weight alkanes under mesophilic, sulfate-reducing conditions: metabolic intermediates and community patterns. FEMS Microbiol. Ecol. 72, 485–495. 10.1111/j.1574-6941.2010.00866.x 20402777

[B69] Singh H. B. O'Hara D. Herlth D. Sachse W. Blake D. R. Bradshaw J. D. (1994). Acetone in the atmosphere: distribution, sources, and sinks. Geophys. Res. 99, 1805–1819. 10.1029/93JD00764

[B70] Stagars M. H. Ruff S. E. Amann R. Knittel K. (2016). High diversity of anaerobic alkane-degrading microbial communities in marine seep sediments based on (1-methylalkyl)succinate synthase genes. Front. Microbiol. 6:1511. 10.3389/fmicb.2015.01511 26779166 PMC4703814

[B71] Von Netzer F. Pilloni G. Kleindienst S. Krüger M. Knittel K. Gründger F.. (2013). Enhanced gene detection assays for fumarate-adding enzymes allow uncovering of anaerobic hydrocarbon degraders in terrestrial and marine systems. Appl. Environ. Microbiol. 79, 543–552. 10.1128/AEM.02362-12 23124238 PMC3553772

[B72] Wang P. Xiao X. Zhang H. Wang F. (2008). Molecular survey of sulphate- reducing bacteria in the deep-sea sediments of the West Pacific warm pool. J. Ocean. Univ. Chin. 7, 269–275. 10.1007/s11802-008-0269-9

[B73] Wawrick B. Mendivelso M. Parisi V. A. Suflita J. M. Davidova I. A. Marks C. R. (2012). Field and laboratory studies on the bioconversion of coal to methane in the San Juan Basin. FEMS Microbiol. Ecol. 81, 26–42. 10.1111/j.1574-6941.2011.01272.x 22146015

[B74] Widdel F. Grundmann O. (2010). Biochemistry of the anaerobic degradation of non-methane alkanes, in Handbook of Hydrocarbon and Lipid Microbiology, ed Timmis K. N. (Berlin: Springer-Verlag), 909–924.

[B75] Wilkes H. Kühner S. Bolm C. Fischer T. Classen A. Widdel F. (2003). Formation of n-alkane- and cycloalkane-derived organic acids during anaerobic growth of a denitrifying bacterium with crude oil. Org. Geochem. 34, 1313–1323. 10.1016/S0146-6380(03)00099-8

[B76] Wilkes H. Rabus R. Fischer T. Armstroff A. Behrends A. Widdel F. (2002). Anaerobic degradation of n-hexane in a denitrifying bacterium: further degradation of the initial intermediate (1-methylpentyl)succinate via C-skeleton rearrangement. Arch. Microbiol. 177, 235–243. 10.1007/s00203-001-0381-3 11907679

[B77] Yakimov M. M. Timmis K. N. Golyshin P. N. (2007). Obligate oil-degrading marine bacteria. Curr. Opin. Biotechnol. 18, 257–266. 10.1016/j.copbio.2007.04.006 17493798

[B78] Zedelius J. Rabus R. Grundmann O. Werner I. Brodkorb D. Schreiber F.. (2011). Alkane degradation under anoxic conditions by a nitrate-reducing bacterium with possible involvement of the electron acceptor in substrate activation. Environ. Microbiol. Rep. 3, 125–135. 10.1111/j.1758-2229.2010.00198.x 21837252 PMC3151549

[B79] Zengler K. Richnow H. H. Rosselló-Mora R. Michaelis W. Widdel F. (1999). Methane formation from long-chain alkanes by anaerobic microorganisms. Nature 401, 266–269. 10.1038/45777 10499582

